# The Sensory and Perceptual Scaffolding of Absorption, Inner Speech, and Self in Psychosis

**DOI:** 10.3389/fpsyt.2021.649808

**Published:** 2021-05-10

**Authors:** Cherise Rosen, Michele Tufano, Clara S. Humpston, Kayla A. Chase, Nev Jones, Amy C. Abramowitz, Ann Franco Chakkalakal, Rajiv P. Sharma

**Affiliations:** ^1^Department of Psychiatry, University of Illinois at Chicago, Chicago, IL, United States; ^2^School of Psychology, Institute for Mental Health, University of Birmingham, Birmingham, United Kingdom; ^3^Department of Psychiatry, University of South Florida, Tampa, FL, United States

**Keywords:** psychosis, inner speech and thought, absorption, phenomenology, network analysis

## Abstract

This study examines the interconnectedness between absorption, inner speech, self, and psychopathology. Absorption involves an intense focus and immersion in mental imagery, sensory/perceptual stimuli, or vivid imagination that involves decreased self-awareness and alterations in consciousness. In psychosis, the dissolution and permeability in the demarcation between self and one's sensory experiences and perceptions, and also between self-other and/or inter-object boundaries alter one's sense of self. Thus, as the individual integrates these changes new “meaning making” or understanding evolves as part of an ongoing inner dialogue and dialogue with others. This study consisted of 117 participants: 81 participants with psychosis and 36 controls. We first conducted a bivariate correlation to elucidate the relationship between absorption and inner speech. We next conducted hierarchical multiple regressions to examine the effect of absorption and inner speech to predict psychopathology. Lastly, we conducted a network analysis and applied extended Bayesian Information Criterion to select the best model. We showed that in both the control and psychosis group dialogic and emotional/motivational types of inner speech were strongly associated with absorption subscales, apart from the aesthetic subscale in the control group which was not significant, while in psychosis, condensed inner speech was uniquely associated with increased imaginative involvement. In psychosis, we also demonstrated that altered consciousness, dialogic, and emotional/motivational inner speech all predicted positive symptoms. In terms of network associations, imaginative involvement was the most central, influential, and most highly predictive node in the model from which all other nodes related to inner speech and psychopathology are connected. This study shows a strong interrelatedness between absorption, inner speech and psychosis thus identifying potentially fertile ground for future research and directions, particularly in the exploration into the underlying construct of imaginative involvement in psychotic symptoms.

## Introduction

“*There is no immaculate perception” Nitetzche* *(**1**)*

The interior world is a dynamic and complex source of sensory and perceptual experiences in which the individual constructs and deconstructs the ontological dimensions of self and environment. Touch (tactile systems), sight (visual systems), hearing (auditory systems), smell (olfactory systems), and taste (gustatory systems) are sensory experiences that do not exist in isolation, but rather are a multisensory construct that are influenced by multiple factors. For example, color perception is shaped by multiple determinants that include biological factors (2), cultural factors (3), emotional factors (4), environmental factors, as shown in the case of the blue-black or white-gold dress (5), and priors (6). Color, for e.g., can also influence tactile (7), auditory (8), olfactory (9, 10), and gustatory sensory systems (11). Perception is the subjective process by which individuals interpret these sensory inputs based on prior knowledge. Consequently, when all influencing factors are considered, subjective sensory experience is different between individuals, resulting in unique perceptual worlds (12).

For many, during the psychosis prodrome, alterations in the alignment of sensory and perceptual experiences emerge. It is within this attenuated state that the primary dimensions of psychosis assemble themselves. Phenomenological descriptions of early psychosis often focus on changes in sensory experiences that are heightened, making it difficult for the individual to filter out irrelevant and unwanted stimuli, while at the same time simultaneously drawing the person into an immersive fractal experience of multisensory and perceptual changes (13, 14). As defined in our earlier work, the construct of absorption “describes a state of immersion in (or capture by) mental imagery or perceptual stimuli and correlates with vivid imagination or fantasy. During a period of intense absorption ‘objects of attention' are hypothesized to ‘acquire an importance and intimacy that are normally reserved for the self and may therefore acquire a self-like quality' (14, 15).” In the early stages of self-disturbance, the pre-reflective and uncanny feeling is interwoven with a new reality yet to be revealed; “….it is ‘as if' I am looking at reality with other eyes, it almost seems as if I am awakening.” (16).

As the sensory and perceptual scaffolding is fundamentally altered, the architectural boundaries of the interior and exterior distinctions deconstruct. The dissolution and permeability of the demarcation between self and one's sensory experiences and perceptions, between self-other and/or inter-object boundaries alter one's sense of self. Thus, as the individual integrates these changes new ‘meaning making' or understanding evolves: “Everything I see is split up. It's like a photograph that's torn in bits and put together again. If somebody moves or speaks, everything I see disappears quickly and I have to put it together” (17–20). These early and complex phenomenological alterations in sensory and perceptual inputs as well as cognitive, bodily, and social experiences are associated with altered self-experience as a core element from which the emergence of psychosis extends (21, 22).

Sense of self and “meaning making” of alterations in sensory and perceptual experiences in psychosis are “dialogical” processes consisting of ongoing inner dialogue/speech and dialogue with others. Inner-speech is defined as a conversation that occurs internally. Inner-speech in participants with psychosis has been shown to be differentially expressed in the presence of sensory and perceptual experiences, when compared to non-clinical controls (23, 24). Theoretically, Dialogical Self Theory (DST), posits self as the narrator in which the “I-position” bridges both inner dialogue/speech with oneself and outer dialogue with others (25, 26). There are multiple forms of inner speech. For example, dialogic inner speech involves an internal conversation with self; condensed inner speech the dialogue is abbreviated; other's voices that occur in inner speech are thoughts heard in the voice of another person; and the evaluative/motivational characteristics of inner speech (27–30). It has been proposed that “the development of human existence is a dialectical process” that hinges on seemingly obvious assumptions or shared “common sense” which in the presence of anomalous experiences the obvious becomes questionable, leaving one's self to make inferences about the experience (31–34). Common sense has been described as “the shared background that makes it possible to live in time without slipping into madness. This background in not so much a collection of statements of knowledge as it is a practice of habits, modes of behavior, and attitudes that together form the substructure, or the framework itself…(35).”

As with the experience of viewing color, the vast heterogeneity of psychosis is also influenced by biological, cultural, emotional, environmental factors, and priors. The primary dimensions of psychosis include the positive symptom dimension of hallucinations, delusions, formal thought disorder; the negative symptom dimension of affect flattening, avolition, and alogia; and the cognitive dimension of disorganization, abstract thinking and attention (36–38). These heterogeneities in psychotic experience and dimensions (e.g., a manic psychotic experience being often markedly different in its phenomenology from a “bizarre delusional state” or traumatogenic voices) are critical to understanding associations between absorption, inner speech and psychosis –almost certainly they interact with (again just for example) a heightened manic-grandiose state in a different way from what would otherwise happen with bizarre delusions which is yet again different from ongoing trauma-related voices or intrusions. This study provides the groundwork by which future studies can further examine the nuances of these interactions.

### Study Aims

The primary aims of this study were to examine the phenomenological construct of sensory and perceptual experiences within the framework of absorption and inner speech in participants experiencing present state psychosis compared to non-clinical controls. We investigated the following questions:

(1) What is the relationship between absorption and inner speech in participants with present-state psychosis compared to a non-clinical control group?

(2) Is there an association between alterations in sensory and perceptual experiences, absorption and inner speech in psychosis?

(3) Does absorption and/or inner speech predict positive, negative or cognitive disorganization in participants with psychosis?

(4) What are the interactions between network components of absorption, inner speech, and psychopathology?

## Methods

### Participants

The University of Illinois at Chicago Internal Review Board (IRB2012-0113) approved the study and all procedures were conducted in accordance with the Declaration of Helsinki (39). All participants gave signed written informed consent prior to the initiation of study procedures.

There were 117 participants in the study for whom all research measures were obtained. The clinical sample consisted of 81 participants, of whom 54 were diagnosed with schizophrenia and 27 were diagnosed with bipolar disorder with psychosis. The non-clinical control group (NCC; *n* = 36) did not meet the Structured Clinical Diagnostic Interview [SCID-IV; (40)] criteria for a current or past major psychiatric disorder. The study exclusion criteria included current substance dependence, seizure disorders, current pregnancy, and neurological conditions. Demographics were obtained at baseline. Duration of untreated psychosis (DUP) was defined as the number of years between onset of psychosis and initiation of antipsychotic medication. Duration of illness was defined as the number of years between onset of illness and current age. IQ was measured using the Wechsler Test of Adult Reading (WTAR) (41).

### Measures Used to Evaluate the Relationship Between Sensory and Perceptual Alterations, Absorption, and Inner Speech

The primary measures of this study aimed to evaluate sensory and perceptual alterations, absorption, and inner speech that included the Positive and Negative Syndrome Scale (PANSS), Tellegen Absorption Scale (TAS), and the Varieties of Inner Speech Questionnaire (VISQ).

### Measure Used to Evaluate Sensory and Perceptual Experiences

Psychopathology was assessed using the Positive and Negative Syndrome Scale (PANSS) (42, 43). PANSS items were scored along a continuum of severity between 1 (asymptomatic) to 7 (extreme symptom severity). Scores were calculated for three-factors: Positive symptoms (POS; “delusions, hallucinatory behavior, suspiciousness or persecution, unusual thought content”), Negative symptoms (NEG; “blunted affect, emotional withdrawal, poor rapport, passive/apathetic social withdrawal, lack of spontaneity and flow of conversation, and active social avoidance”), Cognitive Disorganization (COG; “conceptual disorganization, difficulty in abstract thinking, and poor attention”). Internal consistency for the PANSS subscales used in this study α's were between 0.84 and 0.93.

### Measure Used to Evaluate Absorption

Absorption was measured using the Tellegen Absorption Scale (TAS). The TAS is a 34 item true/false questionnaire designed to measure levels of mental involvement with the object of experience (14, 44, 45). Absorption was formulated in terms of “total attention involving a full commitment of available perceptual, motoric, imaginative and ideational resources to a unified representation of attentional object.” The TAS consists of five subscales that include: synesthesia (TAS_SYN;_ “I find that different odors have different colors”), altered states of consciousness (TAS_ACS_; “I sometimes ‘step outside' my usual self and experience an entirely different state of being”), aesthetic involvement in nature (TAS_AN_; “I like to watch cloud shapes change in the sky”), imaginative involvement (TAS_II_; “I am able to wander off into my thoughts while doing a routine task and actually forget that I am doing the task, and then find a few minutes later that I have completed it”), and extrasensory perception or ESP (TAS_ESP_; “I can often somehow sense the presence of another person before I actually see her/him”). In the current study, internal consistency α's ranged from 0.63 to 0.91 for all TAS subscales and TAS total score.

### Measure Used to Evaluate Varieties of Inner Speech

Varieties of Inner Speech Questionnaire [VISQ; (30)] is an 18-item self-report measure designed to evaluate the experience of inner speech. It measures four varieties of inner speech: dialogic (VISQ_DIS_; “When I am talking to myself about things in my mind, it is like I am going back and forward asking myself questions and then answering them”); condensed (VISQ_CIS_; “I think to myself in words using brief phrases and single words rather than full sentences”); other people (VISQ_OIS_; “I hear other people's actual voices in my head, saying things that they actually once said to me”); and evaluative and motivational characteristics (VISQ_EIS_; “I talk silently to myself telling myself not to do things”). Participants rate applicability of each item using on a 6-point Likert scale, ranging from “Certainly does not apply to me” (1) to “Certainly applies to me” (6). In the current study, internal consistency α's ranged from 0.55 to 0.90 for all VISQ subscales.

### Data Analyses

Statistical analyses were conducted using SPSS version 24.0 and all network analyses were conducting using the statistical programming language R in RStudio (Version 1.2.5033), using the mgm package (version 1.2–7), qgraph package (version 1.6.5), and bootnet package (version 1.4.3), as reported in Pappa et al. (46). Demographic data were analyzed using Chi-square tests to measure differences between groups in sex and race, analyses of variance (ANOVAs) was applied to measure differences in age and IQ between groups and independent sample *t*-tests were conducted to measure differences between the onset of illness, duration of untreated psychosis and duration of illness between schizophrenia and bipolar with psychosis groups. ANOVAs that yielded significant results were followed up with Bonferroni *post-hoc* tests to identify significant pair-wise group differences. Pearson's bivariate correlations with bootstrapping at 1,000 iterations were conducted to determine associations between TAS and VISQ subscales in the clinical and non-clinical groups. Hierarchical multiple linear regressions were used to examine the unique contribution of absorption and inner speech in the prediction of positive, negative, and cognitive disorganization in participants with present state psychosis. A two-step hierarchical multiple linear regression model was performed to predict the presence and severity of PANSS positive, negative, and cognitive disorganization subscale scores examining the relative contribution of the TAS scores (synesthesia, altered states of consciousness, aesthetic, imaginative, and ESP), and VISQ subscale scores (condensed inner speech, evaluative and motivational inner speech, and dialogic inner speech). There were a total of 81 subjects included in the analysis. The relevant assumptions of this statistical analysis were tested (47). The collinearity statistics were within accepted limits (48). Mahalanobis distance scores indicated no multivariate outliers. Residual and scatter plots indicated that the assumptions of normality, linearity and homoscedasticity were all satisfied (48).

Lastly, for the network analysis, we used the mgm package (version 1.2–7) to estimate the network model (49). The Mixed Graphical Models (MGMs) allow modeling of cross-sectional data with ℓ_1_-regularized (LASSO) neighborhood regression, which facilitates model interpretation by attributing the zero value to artificial edge-parameters (50, 51). We used Extended Bayesian Information Criterion to select the best model (52). The stability and reliability of the network model and its centrality indices were assessed with bootstrap method using the *bootnet* package. The network model and centrality indices were displayed using the *qgraph* package (53, 54).

## Results

### Descriptive Characteristics

Group comparisons of demographics revealed no statistically significant difference between sex, race, age and IQ between the clinical and NCC groups (See Table 1). There was no significant difference between clinical groups regarding age of onset of illness, duration of untreated psychosis and duration of illness. Thus, the clinical sample was combined for the remaining analysis.

**Table 1:** Demographic characteristics of sample (*n* = 117).

	**Control**		**Schizophrenia**		**Bipolar with**		**Group difference**
	**(*n* = 36)**		**(*n* = 54)**		**psychosis**		
					**(*n* = 27)**		
Sex							$\chi_{\left(2\right)}^{2}$ = 0.23, *p* = 0.89
Male	19 (53%)		29 (54%)		13 (48%)		
Female	17 (47%)		25 (46%)		14 (52%)		
Race							$\chi_{\left(6\right)}^{2}$ = 8.69, *p* = 0.19
African American	25 (69%)		42 (78%)		22 (82%)		
Asian	3 (8%)		0 (0%)		0 (0%)		
Caucasian	7 (19%)		8 (15%)		3 (11%)		
Hispanic	1 (3%)		4 (7%)		2 (7%)		
	**Mean**	**SD**	**Mean**	**SD**	**Mean**	**SD**	
Age	44.22	14.20	47.28	10.48	45.85	12.11	*F*_(2, 114)_ = 0.69, *p* = 0.50
IQ	96.19	14.40	92.69	10.69	92.47	9.23	*F*_(2, 98)_ = 1.00, *p* = 0.37
Age at onset of illness			19.54	7.05	21.68	8.01	*t*_(77)_ = −1.20, *p* = 0.23
Duration of untreated psychosis			5.34	7.18	4.11	9.09	*t*_(75)_ = 0.63, *p* = 0.53
Duration of illness			27.74	12.64	23.56	14.24	*t*_(77)_ = 0.26, *p* = 1.31

*SD, Standard deviation*.

### Relationship Between Absorption and Inner Speech in Participants With Present-State Psychosis and Non-clinical Control Group

To elucidate the relationship between absorption and inner speech, we conducted a bivariate correlation with confidence intervals calculated at 1,000 iterations bootstrapped and found multiple positive associations. As reported in Supplementary Table 1, the combined sample associations were found in all domains between absorption and inner speech with the exception of VISQ_CIS_ which was only associated with TAS_II._

**Figure 1:**
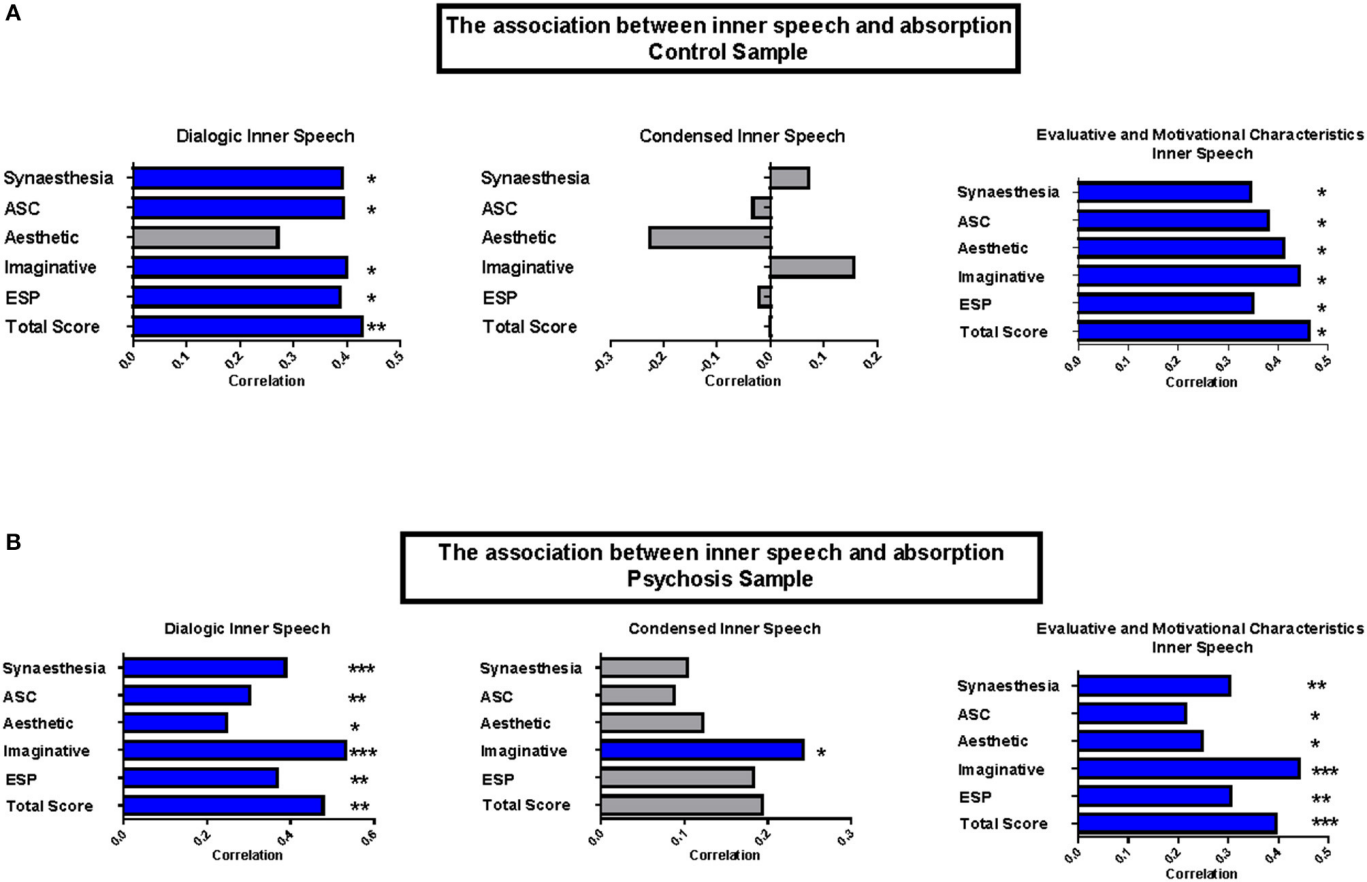
Association between inner speech and absorption. Pearson's r correlations (95% confidence intervals) between Absorption and VISQ subscales. Confidence intervals calculated using 1,000 iterations bootstrapped sample. *p<0.05; **p<0.01; ***p<0.001 (Blue bars indicate statistical significance). **(A)** The association between inner speech and absorption control sample. **(B)** The association between inner speech and absorption psychosis sample.

As shown in Figure 1A, the non-clinical sample shows a positive association between VISQ_DIS_ and TAS_SYN_, TAS_ACS_, TAS_AN_, TAS_II_, and TAS_ESP_. Likewise, VISQ_EIS_ also showed a positive correlation with TAS_SYN_, TAS_ACS_, TAS_AN_, TAS_II_, and TAS_ESP_. However, there was no association between VISQ_CIS_ and any of the TAS subscales.

The clinical sample shared a similar pattern, as shown in Figure 1B, in that there was a positive association between VISQ_DIS_ and TAS_SYN_, TAS_ACS_, TAS_AN_, TAS_II_, and TAS_ESP._ VISQ_EIS_ also showed a positive association with TAS_SYN_, TAS_ACS_, TAS_AN_, TAS_II_, and TAS_ESP_. Uniquely, VISQ_CIS_ in the clinical sample was associated with TAS_II_.

### Predictive Dimensions of Absorption and Inner Speech With Positive, Negative, and Cognitive Disorganization in Participants With Psychosis

We conducted a hierarchical multiple regression to examine the effect of absorption and varieties of inner speech to predict increased levels of positive, negative, and cognitive disorganization symptoms in participants with psychosis (*n* = 81). For the purposes of this analysis, other people inner speech was removed from the analysis due to the potential confound between other people inner speech and internal auditory verbal hallucinations.

We first examined the predictive power of absorption and inner speech with positive symptoms as the dependent variable. In step one of the model, we entered aspects of absorption, as measured by TAS subscales; in step two of the model, we entered the three VISQ subscale scores. Hierarchical multiple regression indicated that at step one, the TAS_ASC_ subscale scores contributed significantly to the regression model [*F*_(5,75)_ = 2.69, *p* = 0.03, *R*^2^ = 0.152]. In step two of the model we entered the three VISQ subscale scores, which further significantly contributed to the model [Δ*F*_(3,72)_ = 4.27, *p* ≤ 0.008, Δ*R*^2^ = 0.13]. The overall model was significant [*F*_(8,72)_ = 3.504, *p* =0.002, *R*^2^ = 0.28]. When all independent variables were included in the regression model, TAS_ACS_ (*p* = 0.03), VISQ_DIS_ (*p* = 0.01), and VISQ_CIS_ (*p* = 0.05) scores significantly predicted increased psychotic symptoms and this accounted for 28% of the variance in positive symptoms scores (See Supplementary Table 2).

We next examined the predictive power of absorption and inner speech with negative symptoms as the dependent variable. In step one, we entered subscale scores of absorption; in step two, we entered the VISQ subscale scores. The hierarchical multiple regression showed that at step one, absorption scores did not contribute significantly to the regression model [*F*_(5,75)_ = 1.27, *p* = 0.29, *R*^2^ = 0.078]. In step two of the model we entered the three VISQ subscale scores, which also did not significantly contribute to the model [Δ*F*_(3,72)_ = 2.31, *p* = 0.08, Δ*R*^2^ = 0.08]. The overall model was not significant [*F*_(8,72)_ = 1.70, *p* = 0.011, *R*^2^ = 0.16].

Lastly, we examined the power of absorption and inner speech in predicting increased cognitive disorganization. In step one, we entered aspects of absorption as measured by TAS subscales; in step two, we entered the three VISQ subscale scores. The hierarchical multiple regression revealed that at step-one, TAS_ESP_ (*p* = 0.008) scores contributed significantly to the regression model [*F*_(5,75)_ = 2.5, *p* = 0.04, *R*^2^ = 0.14]. In step two of the model we entered the three VISQ subscale scores, which significantly contributed to the model [Δ*F*_(3,73)_ = 4.06, *p* =0.01, Δ*R*^2^ = 0.12]. The overall model was significant [*F*_(8,72)_ = 3.28, *p* = 0.003, *R*^2^ = 0.27]. When all independent variables were included in the regression model, TAS_ESP_ (*p* = 0.01), VISQ_DIS_ (*p* = 0.001), and VISQ_EIS_ (*p* = 0.01) scores significantly predicted increased absorption and this model accounted for 27% of the variation in cognitive disorganization scores (See Supplementary Table 3).

### Interactive Network Components of Absorption, Inner Speech, and Psychopathology

**Figure 2:**
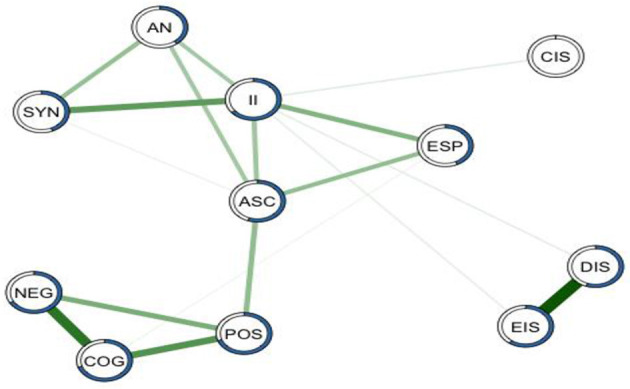
Interactive network for the components of absorption, inner speech, and psychopathology. MGM predictability interactive network for the components of absorption, inner speech, and psychopathology. Each node represents a variable, including aesthetic nature (AN), synesthesia (SYN), imaginative involvement (II), altered states of consciousness (ASC), extra sensory perception (ESP), negative symptoms (NEG), cognitive disorganization (COG), positive symptoms (POS), condensed inner speech (CIS), evaluative and motivational characteristics (EIS), and dialogic inner speech (DIS). Green lines connecting nodes symbolizes positive interactions, with the width of the edges proportional to the strength of each interaction. The proportion of each node's variance explained by all the other nodes in the network is shown by blue rings surrounding each node.

The main goal of this network analysis was to explore the centrality of the constructs of absorption, inner speech and psychopathology to potentially identify core features of interrelatedness. Network analysis uses the graph theory to analyze individual symptoms (paranoia, hallucinations, or depression) or constructs such as absorption, inner speech and psychopathology to reveal the most influential underlying structure (46, 55–57). The network structure of absorption, inner speech, and psychopathology are presented in Figure 2. In the network model, each variable is represented by a node (illustrated as a dot), while each interaction among the nodes is represented by an edge (illustrated as a line). The probability of each node is calculated by estimating the nodes connecting to it (nodewise predictability). Additionally, the predictability of each node within the network is calculated. In this way, this method augments the reliability of the network structure (50). Nodes within the networks were determined using the force-directed Fruchterman-Reingold algorithm, which tends to group stronger nodes connections at the center of the network, in order to avoid edge crossing (58). Positive and negative interactions among nodes are displayed using the *qgraph* package and are represented in green and red edges, respectively, with the width of the edges proportional to the strength of each interaction. However, all the interactions among the nodes of the network are positive, thus no red edges/negative interactions are represented.

All nodes are included in the overall network structure. The contribution of condensed inner speech to the network structure is minimal. Psychopathology (cognitive disorganization, positive symptoms and negative symptoms) was most predictive of the network model of absorption (TAS_II_, TAS_ACS_); absorption and inner speech (VISQ_EIS_,VISQ_DIS_) were also closely related with each other (Supplementary Table 4).

**Figure 3:**
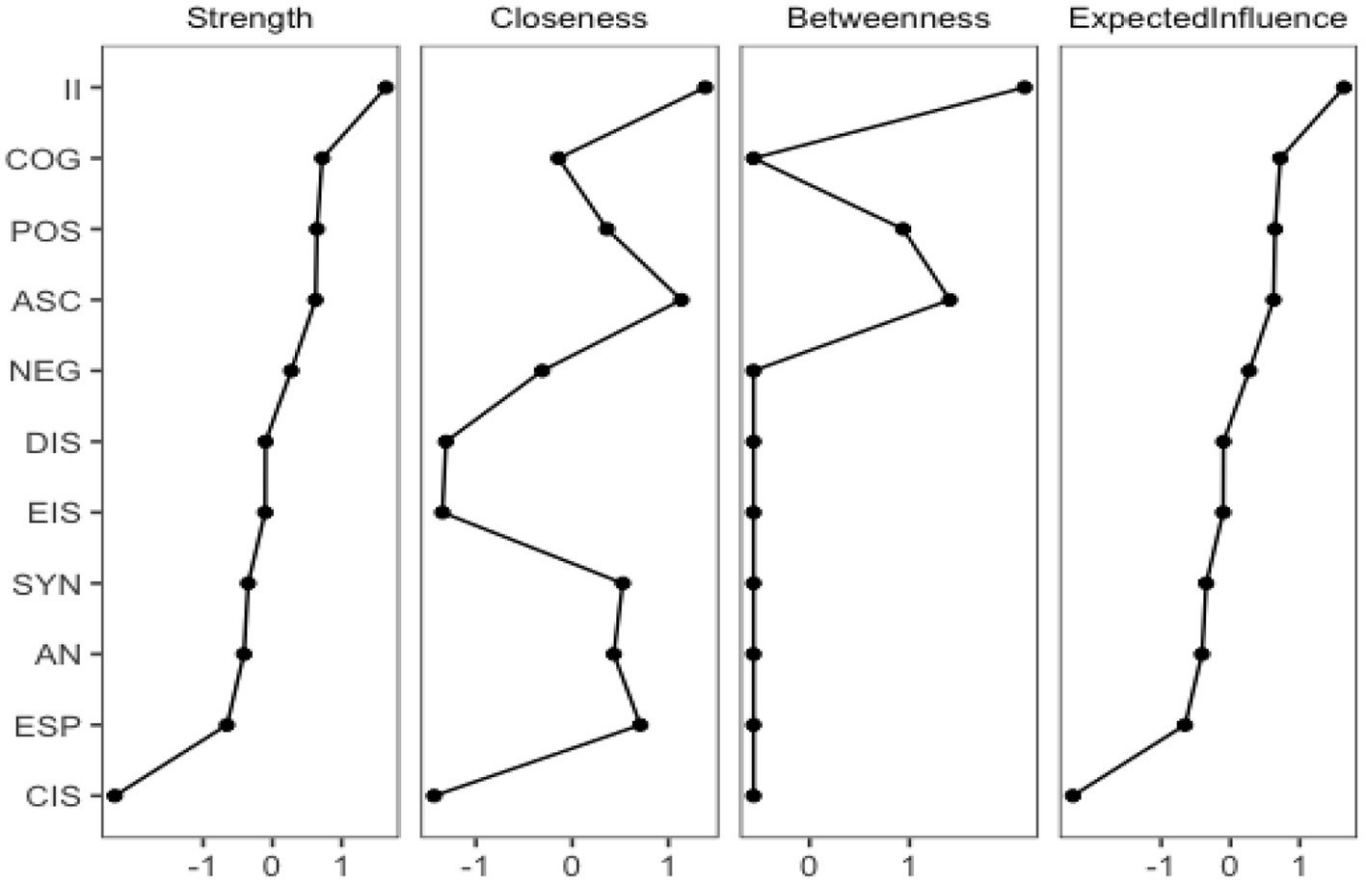
Centrality metrics intervals for the interactive network components of absorption, inner dialogue, and psychopathology. Centrality metrics intervals for the interactive network components of absorption, inner speech, and psychopathology, ordered according to the Expected Influence. II, Imaginative involvement; COG, cognitive disorganization; POS, positive symptoms; ASC, altered states of consciousness; NEG, negative symptoms; DIS, dialogic inner speech; EIS, evaluative and motivational characteristics; SYN, synesthesia; AN, aesthetic nature; ESP, extra sensory perception; CIS, condensed inner speech.

Indices included in the centrality analysis as noted in Figure 3 were: *closeness*, which quantifies the indirect strength of connection between a node to other nodes; *betweenness*, which quantifies the importance of each node for the interaction between two adjacent nodes; *node strength*, which is the sum of weighted correlation coefficient of all the interactions connected to a single node (54); and *expected influence*, which takes into account the negative edges to evaluate the strength and influence of each node within a network (59).

Interestingly imaginative involvement (TAS_II_) was found to be the most central, influential, and most highly predictive node in the model from which all other nodes are connected. The proximity pathways of the nodes most closely related are TAS_II_ (1.38), altered states of consciousness (TAS_ACS_ = 1.14), and positive symptoms (POS = 0.36). The nodes that predict the network model the most within the expected influence pathway are TAS_II_ (1.64), COG (0.72), POS (0.74), and TAS_ACS_ (0.62).

## Discussion

Think back to a time when you heard your name being called from across the room when no one was there, caught a fleeting image or shadow in the corner of your vision when nothing was there, felt a light sensation brush across your skin in the absence of external stimuli, or even briefly experienced a scent that could not be associated with the environment. Think about the shifting of salience you gave the experience, the feelings that accompanied the experience, the inner speech as your mind stacked up all the priors to make sense of the experience before you categorized it. Now imagine that the frequency, duration, and intensity of the experience increase, and thoughts turn inward in the search to reconstruct equilibrium. Alterations in the process of integrating sensory and perceptual experiences in psychosis are thought to be closely associated to the phenomenon of absorption or aberrant salience (60). The etiology of alterations in sensory and perceptual experiences fundamentally consist of an association between absorption, inner speech and dimensions of psychopathology that are not under direct or voluntary control, all of which are rooted in interactions between complex cultural, environmental, biological, and psychological substrates (33, 61, 62). In this paper we examined the associations between absorption, inner speech, and psychopathology in relation to self within the psychotic experience.

Our previous research has shown that absorption is more prevalent and intense in participants with psychosis when compared to non-clinical controls, and was significantly associated with hallucinations and delusions (15). We have also previously shown that dialogic inner speech is associated with psychosis (24). In this study we report that in participants with psychosis, dialogic inner speech and evaluative and motivational inner speech were associated with all factors of absorption whereas condensed inner speech was associated with imaginative involvement. We also report that altered states of consciousness, dialogic inner speech, and condensed inner speech predict increased positive symptoms; and that extrasensory perception, dialogic inner speech and evaluative and motivational inner speech predict both increased cognitive disorganization and negative symptoms. Other studies have shown that TAS and nonclinical psychotic assessments were structurally distinct (63). In terms of network associations and pathways, imaginative involvement was found to be the most central, influential, and most highly predictive node in the model from which all other nodes related to psychopathology and inner speech are connected.

Absorption is defined as a process by which the individual is immersed in mental imagery or perceptual stimuli, vivid imagination or fantasy. During a period of intense absorption “objects of […] attention” are believed to “acquire an importance and intimacy that are normally reserved for the self and may therefore acquire a self-like quality” (14, 64). Absorption is also associated with decreased self-awareness and increased alteration in state of consciousness (65). Intense absorption is closely related to basic self-disturbances, which are expressed in the dissolution of the boundaries between self and the interior and external worlds (66–68). Alterations in sensory and perceptual experiences related to changes in self-experience are supported by phenomenological, experimental and neurophysiological data that suggest possible neurobiological underpinnings of a core disturbance (22, 69, 70). For example, the core network node, imaginative involvement, includes the endorsement of items along themes of losing connections to self and others while becoming internally immersed where one becomes oblivious to one's environment, experiencing thoughts as visual images, and attributing meaning to something that is otherwise meaningless. Imaginative involvement has been described as an element of absorption where the individual may become vividly immersed in their imagination that at times it can become difficult to distinguish imagination from reality (71). Additionally, imaginative involvement can include the experience of “loss of time” or the feeling of performing in “auto-pilot,” which have been linked to automatic features of dissociation that can also be experienced in psychosis: “I am able to wander off into my thoughts while doing a routine task and actually forget that I am doing the task, and them find a few minutes later that I have completed it” (72, 73). The nodes most closely related to imaginative involvement are altered states of consciousness and positive symptoms. This central pathway further supports the blueprint of interconnection between alterations in sense of self, as is seen in basic symptoms, increased absorption and psychosis.

The network node imaginative involvement is also loosely associated to dialogic inner speech, evaluative and motivational inner speech, and condensed inner speech. Yet the association between absorption and inner speech in psychosis demonstrates that all factors of absorption, including imaginative involvement, were significantly associated with dialogic inner speech, evaluative and motivational inner speech. Imaginative involvement was also significantly associated with condensed inner speech. Inner speech appears to be connected to positive symptoms via the imaginative involvement/altered states of consciousness pathway, suggesting interconnectedness between inner speech, particularly dialogic inner speech when strongly associated with evaluative and motivational inner speech, and positive symptoms as part of the imaginative involvement and altered states of consciousness pathway. Kusters (35) eloquently describes the experience by which thoughts within the imaginative process are given life: “You think about something, and at the same time you think about everything that surrounds what you're thinking about. You think under it, over it, and behind it. With paranoia, thinking is the same as creating. A theoretical point of concentration expands into a practical creative field. By thinking somewhere behind it, you even change the character of the object of the thought itself. By imagining something, you bring it to life (35).”

### Limitations

This study has several limitations. First, the sample size is modest and there is only limited generalizability of these results as the data are largely based on self-report. Second, as culture significantly influences the subjective experience of psychosis, our results may not generalize to other regions and populations. Third, all measures were collected concurrently, limiting any causal inferences of these associations and pathways. For example, many people with chronic and persistent psychosis may become socially isolated, which could influence both inner speech and introspection and absorption. Also, if one is experiencing profound alterations of perception, and starts to look for reasons, this again may fuel introspection, immersive attention and inner speech. Thus, prospective longitudinal research would be critical to unpacking the complex ways in which inner speech might be a building block of psychosis, but could also go the reverse way, and likewise for absorption. Additionally, a general limitation of network analysis is the difficulty to rule out that the findings are not simply related to measurement-related issues, such as the way subscales of one instruments will tend to covey more with one another than with subscales of other instruments due to issues like scoring range similarities or conceptual similarities. Lastly, the dimensions of absorption in the TAS can directly overlap with psychosis, thus classic psychotic symptoms, for example, can be structurally similar to the TAS dimensions/sub-scales which can be seen as both a limitation (of measurement, primarily; of psychometric validity especially across populations) and strength as potentially fertile ground for future research directions.

## Conclusion

This study examined the relationship between absorption, inner speech, and psychopathology. In psychosis, dialogic inner speech and emotional/motivational inner speech are strongly associated with all dimensions/subscales of absorption while condensed inner speech is singularly associated with increased imaginative involvement. We also demonstrated that altered states of consciousness, dialogic inner speech, and emotional/motivational inner speech all predicted positive symptoms. In terms of network associations and pathways within the scaffolding of absorption and inner speech in psychosis, imaginative involvement is the most central, influential, and most highly predictive node in the model from which all other nodes related to psychopathology and inner speech are connected. This interconnectedness has been described as if you were trying to grasp water, as if you had burnt your tongue in a flame, as if time had evaporated and condensed into fantasy. Something strange occurred, but how it occurred, what it was, and whether it was real or not—that all remains vague. You walk through the mirror, stroll around behind it a bit and come back; “in front of” turns out to have been “behind” and vice versa…(35). Lastly, this study shows that within the network of absorption, inner speech and psychopathology, vivid sensory, and perceptual imaginative involvement that encompasses dialogic inner speech with evaluative and motivational characteristics as expressed in alterations of consciousness is an interconnected structure in psychotic experiences.

## Data Availability Statement

The datasets presented in this article are not readily available because individual raw data cannot be made publicly available as ethical consent was not provided by participants nor do we have IRB approval to do such. Requests to access the datasets should be directed to Cherise Rosen, ccrosen@uic.edu.

## Ethics Statement

The study presented involved human participants and was reviewed and approved by the University of Illinois at Chicago Internal Review Board. All participants provided their written informed consent to participate in this study prior to study initiation.

## Author Contributions

CR and RS contributed to the conception and design of the study. CR and KC were involved in data collection, data analysis, and interpretation. MT conducted the network analysis and MT and CR contributed to the interpretation. CR, CH, KC, AA, and AF contributed to the drafting the article. CR, CH, KC, NJ, and RS contributed to the critical revision of the article. All authors have given final approval of the version to be published.

## Conflict of Interest

The authors declare that the research was conducted in the absence of any commercial or financial relationships that could be construed as a potential conflict of interest.
