# Supplementary material for: The Sensory and Perceptual Scaffolding of Absorption, Inner Speech, and Self in Psychosis
Source: Front Psychiatry. 2021 May 10;12:649808. doi: 10.3389/fpsyt.2021.649808 (PMC8145281; doi:10.3389/fpsyt.2021.649808)
Supplement: Supplementary file 1 [file Table_1.docx]

Supplemental Table 1 (Full sample associations, n=117)

|  | VISQ_DIS_ | VISQ_CIS_ | VISQ_OIS_ | VISQ_EIS_ |
| --- | --- | --- | --- | --- |
| Synesthesia  (TAS_SYN_) | 0.379 [.236, .507]  ***p* ≤ 0.00** | 0.093 [-.080, .254]  *p* = 0.32 | 0.321 [.154, .492]  ***p* ≤ 0.00** | 0.311 [.151, .455]  ***p* = 0.001** |
| Altered states of consciousness (TAS_ACS_) | 0.388 [.253, .523]  ***p* ≤ 0.00** | 0.073 [-.109, .237]  *p* = 0.43 | 0.465 [.307, .598]  ***p* ≤ 0.00** | 0.326 [.170, .462]  ***p* ≤ 0.00** |
| Aesthetic involvement in nature (TAS_AN_) | 0.243 [.083, .387]  ***p* = 0.008** | -0.003 [-.183, .169]  *p* = 0.98 | 0.205 [.007, .393]  ***p* = 0.03** | 0.297 [.129, .446]  ***p* = 0.001** |
| Imaginative involvement (TAS_II_) | 0.506 [.353,.632]  ***p* ≤ 0.00** | 0.215 [.043, .384]  ***p* = 0.02** | 0.460 [.300, .600]  ***p* ≤ 0.00** | 0.468 [.306, .599]  ***p* ≤ 0.00** |
| ESP (TAS_ESP_) | 0.407 [.259,.542]  ***p* ≤ 0.00** | 0.127 [-.041, .297]  *p* = 0.17 | 0.448 [.302, .601]  ***p* ≤ 0.00** | 0.356 [.200, .498]  ***p* ≤ 0.00** |
| Total Score | 0.488 [.344,.608]  ***p* ≤ 0.00** | 0.131 [-.033, .295]  *p* = 0.16 | 0.482 [.326, .613]  ***p* ≤ 0.00** | 0.452 [.284, .586]  ***p* ≤ 0.00** |

Note. Confidence intervals calculated using 1000 iterations bootstrapped samples. VISQ_DIS_ = Dialogic inner speech.

VISQ_CIS_ = Condensed inner speech. VISQ_OIS_ = Other people inner speech. VISQ_EIS_ = Evaluative and motivational inner speech
