# Supplementary material for: The Sensory and Perceptual Scaffolding of Absorption, Inner Speech, and Self in Psychosis
Source: Front Psychiatry. 2021 May 10;12:649808. doi: 10.3389/fpsyt.2021.649808 (PMC8145281; doi:10.3389/fpsyt.2021.649808)
Supplement: Supplementary file 2 [file Table_2.docx]

Supplemental Table 2: Regression analyses predicting Positive symptoms scores (n=81)

| **Step** | **Variables entered** | ***B*** | ***SE*** | **β** | ***t*** | ***p*** | ***VIF*** |
| --- | --- | --- | --- | --- | --- | --- | --- |
| 1 | Synesthesia | -.330 | .556 | -.085 | -.594 | .554 | 1.803 |
|  | **Altered states of consciousness** | **1.023** | **.481** | **.298** | **2.127** | **.037** | **1.732** |
|  | Aesthetic | -.249 | .472 | -.076 | -.528 | .599 | 1.827 |
|  | Imaginative | .103 | .308 | .051 | .334 | .739 | 2.044 |
|  | ESP | .803 | .541 | .194 | 1.483 | .142 | 1.507 |
|  | | | | | | | |
| 2 | Synesthesia | -.504 | .529 | -.129 | -.952 | .344 | 1.847 |
|  | **Altered states of consciousness** | **1.044** | **.456** | **.304** | **2.289** | **.025** | **1.760** |
|  | Aesthetic | -.070 | .450 | -.021 | -.156 | .876 | 1.880 |
|  | Imaginative | -.285 | .314 | -.141 | -.907 | .367 | 2.401 |
|  | ESP | .499 | .518 | .120 | .964 | .338 | 1.557 |
|  | **VISQ_DIS_** | **.378** | **.147** | **.427** | **2.565** | **.012** | **2.773** |
|  | **VISQ_CIS_** | **.177** | **.089** | **.208** | **1.986** | **.051** | **1.094** |
|  | VISQ_EIS_ | -.116 | .147 | -.123 | -.792 | .431 | 2.425 |

Note. VISQ_DIS_ = Dialogic inner speech VISQ subscale. VISQ_CIS_ = Condensed inner speech VISQ subscale. VISQ_EIS_ = Evaluative and motivational inner speech VISQ subscale
