# Supplementary material for: The Sensory and Perceptual Scaffolding of Absorption, Inner Speech, and Self in Psychosis
Source: Front Psychiatry. 2021 May 10;12:649808. doi: 10.3389/fpsyt.2021.649808 (PMC8145281; doi:10.3389/fpsyt.2021.649808)
Supplement: Supplementary file 3 [file Table_3.docx]

Supplemental Table 3: Regression analyses predicting Cognitive Disorganization symptoms scores (n=81)

| **Step** | **Variables entered** | ***B*** | ***SE*** | **β** | ***t*** | ***p*** | ***VIF*** |
| --- | --- | --- | --- | --- | --- | --- | --- |
| 1 | Synesthesia | .077 | .344 | .032 | .223 | .824 | 1.803 |
|  | Altered states of consciousness | -.186 | .298 | -.088 | -.626 | .533 | 1.732 |
|  | Aesthetic | .214 | .292 | .106 | .734 | .465 | 1.827 |
|  | Imaginative | -.021 | .191 | -.017 | -.111 | .912 | 2.044 |
|  | **ESP** | **.918** | **.335** | **.359** | **2.738** | **.008** | **1.507** |
|  | | | | | | | |
| 2 | Synesthesia | -.053 | .329 | -.022 | -.162 | .872 | 1.847 |
|  | Altered states of consciousness | -.251 | .283 | -.119 | -.886 | .379 | 1.760 |
|  | Aesthetic | .366 | .280 | .181 | 1.311 | .194 | 1.880 |
|  | Imaginative | -.150 | .195 | -.121 | -.771 | .443 | 2.401 |
|  | **ESP** | **.814** | **.322** | **.319** | **2.532** | **.014** | **1.557** |
|  | **VISQ_DIS_** | **.308** | **.092** | **.565** | **3.362** | **.001** | **2.773** |
|  | **VISQ_CIS_** | .029 | .055 | .055 | .518 | .606 | 1.094 |
|  | **VISQ_EIS_** | **-.238** | **.091** | **-.410** | **-2.611** | **.011** | **2.425** |

Note. VISQ_DIS_ = Dialogic inner speech VISQ subscale. VISQ_CIS_ = Condensed inner speech VISQ subscale. VISQ_EIS_ = Evaluative and motivational inner speech VISQ subscale
