# Supplementary material for: The Sensory and Perceptual Scaffolding of Absorption, Inner Speech, and Self in Psychosis
Source: Front Psychiatry. 2021 May 10;12:649808. doi: 10.3389/fpsyt.2021.649808 (PMC8145281; doi:10.3389/fpsyt.2021.649808)
Supplement: Supplementary file 4 [file Table_4.docx]

Supplemental Table 4: Predictability estimates for the variables present in the interactive network for the components of absorption, inner speech, and psychopathology.

|  | Variable | R2 |
| --- | --- | --- |
| 1 | SYN | 0.434 |
| 2 | ASC | 0.556 |
| 3 | AN | 0.396 |
| 4 | II | 0.643 |
| 5 | ESP | 0.429 |
| 6 | DIS | 0.555 |
| 7 | CIS | 0 |
| 8 | EIS | 0.6 |
| 9 | POS | 0.695 |
| 10 | NEG | 0.668 |
| 11 | COG | 0.698 |

Note. Abbreviations: Synesthesia (SYN), altered states of consciousness (ASC), aesthetic nature (AN), imaginative involvement (II), extra sensory perception (ESP), dialogic inner speech (DIS), condensed inner speech (CIS), evaluative and motivational characteristics (EIS), positive symptoms (POS), negative symptoms (NEG), cognitive disorganization (COG).
